# Comprehensive Assessment of *CFTR* Modulators’ Therapeutic Efficiency for N1303K Variant

**DOI:** 10.3390/ijms25052770

**Published:** 2024-02-27

**Authors:** Anna Efremova, Nataliya Kashirskaya, Stanislav Krasovskiy, Yuliya Melyanovskaya, Maria Krasnova, Diana Mokrousova, Nataliya Bulatenko, Elena Kondratyeva, Oleg Makhnach, Tatiana Bukharova, Rena Zinchenko, Sergey Kutsev, Dmitry Goldshtein

**Affiliations:** 1 Research Centre for Medical Genetics, Moscow 115522, Russia; kashirskayanj@med-gen.ru (N.K.); sa_krasovsky@mail.ru (S.K.); melcat@mail.ru (Y.M.); krasnova.m.g.0605@gmail.com (M.K.); diana-mok2000@yandex.ru (D.M.); natasha.bulatenko@yandex.ru (N.B.); elenafpk@mail.ru (E.K.); buben6@yandex.ru (O.M.); renazinchenko@mail.ru (R.Z.); kutsev@mail.ru (S.K.); dvgoldshtein@gmail.com (D.G.); 2 Moscow Regional Research and Clinical Institute (“MONIKI”), Moscow 129110, Russia; 3 Pulmonology Scientific Research Institute under Federal Medical and Biological Agency of Russian Federation, Moscow 115682, Russia

**Keywords:** cystic fibrosis, N1303K, intestinal current measurements, intestinal organoids, clinical status, FEV_1_, BMI

## Abstract

p.Asn1303Lys (N1303K) is a common missense variant of the *CFTR* gene, causing cystic fibrosis (CF). In this study, we initially evaluated the influence of *CFTR* modulators on the restoration of N1303K-*CFTR* function using intestinal organoids derived from four CF patients expressing the N1303K variant. The forskolin-induced swelling assay in organoids offered valuable insights about the beneficial effects of VX-770 + VX-661 + VX-445 (Elexacaftor + Tezacaftor + Ivacaftor, ETI) on N1303K-*CFTR* function restoration and about discouraging the prescription of VX-770 + VX-809 (Ivacaftor + Lumacaftor) or VX-770 + VX-661 (Ivacaftor + Tezacaftor) therapy for N1303K/class I patients. Then, a comprehensive assessment was conducted on an example of one patient with the N1303K/class I genotype to examine the ETI effect on the restoration of N1303K-*CFTR* function using in vitro the patient’s intestinal organoids, ex vivo the intestinal current measurements (ICM) method and assessment of the clinical status before and after targeted therapy. All obtained results are consistent with each other and have proven the effectiveness of ETI for the N1303K variant. ETI produced a significant positive effect on forskolin-induced swelling in N1303K/class I organoids indicating functional improvement of the *CFTR* protein; ICM demonstrated that ETI therapy restored *CFTR* function in the intestinal epithelium after three months of treatment, and the patient improved his clinical status and lung function, increased his body mass index (BMI) and reduced the lung pathogenic flora diversity, surprisingly without improving the sweat test results.

## 1. Introduction

Cystic fibrosis (CF) is an autosomal-recessive disease that affects one in two thousand five hundred newborn Caucasians, though its frequency may vary in certain groups. CF is caused by the presence of pathogenic variants of the *CFTR* (Cystic Fibrosis Transmembrane Conductance Regulator) gene that encodes 1480 amino acids transmembrane protein and forms cAMP-dependent chloride channel expressed by epithelial cell membranes. CF primarily affects the epithelium of the intestine, the respiratory tract, pancreas, hepatobiliary tract and sweat glands. Poor ciliary clearance with excessive mucus production causes obstructive lung disease and chronic bacterial infections, ultimately leading to respiratory failure, which is the most frequent cause of death among CF patients [1,2].

Target drugs have been used to treat CF since 2012, directly affecting the disease process, increasing the life expectancy of patients and significantly improving life quality. The effectiveness of target drugs varies due to *CFTR* allelic heterogeneity. Currently, Ivacaftor (“Kalydeco”^®^, VX-770) is prescribed in the presence of one out of ninety-seven variants of *CFTR*, while more than 2000 variants are known [3,4]. For Tezacaftor + Ivacaftor (“Symdeko”^®^, VX-661 + VX-770), there were found 154 therapy-susceptible *CFTR* variants and 177 variants susceptive for Elexacaftor + Tezacaftor + Ivacaftor (ETI, “Trikafta”^®^, VX-445 + VX-661 + VX-770). Lumacaftor + Ivacaftor (“Orkambi”^®^, VX-809 + VX-770) is a primary drug of choice for homozygous F508del patients [4,5]. The FDA is considering expanding the list of pathogenic variants in view of positive in vitro targeted drug screening results [6].

Intestinal 3D organoids, derived from the patient’s own intestinal tissue offer unique opportunities for personalized *CFTR* genotype-dependent assessment of target drug efficiency. Intestinal organoids are multicellular 3D spheroids consisting of a single-layered epithelium and represent an in vitro-derived simplified intestine model [7]. Forskolin-induced swelling (FIS) assay using patient-derived intestinal organoids is a simple and convenient method for the determination of the *CFTR* channel function with any *CFTR* genetic variants. This approach is based on organoid swelling upon forskolin stimulation and depends solely on residual *CFTR* chloride channel function [8,9]. Intestinal organoids have been used for CF studies since 2015 [10]. The intestinal organoids method without clinical trials made it possible to study the pathogenicity of *CFTR* complex alleles [11] and several common and rare *CFTR* variants [12,13,14,15] and to determine indications for target drug therapy for CF patients.

The pathogenic variant p.Asn1303Lys (legacy, N1303K) is the tenth most common mutation, with a frequency of 1.53% according to the CF Patient Registry in the Russian Federation in 2020 [16]. According to the European CF Registry (40 countries) in 2021, N1303K occupies third place in terms of its occurrence, with a total incidence of 2.76% and the highest incidence observed in Iceland (46.4%) [17]. In the US, this variant occupies fifth place, with an incidence of 2.4% [18]. Position 1303 in the *CFTR* protein is located in the second nucleotide binding domain (NBD2). Normally, N1303 interacts with glutamine residue 1291 to form a Q-loop in NBD2 [19], and it was shown that the replacement of asparagine by lysine at this position disrupts protein processing and transport [20,21]. Moreover, N1303K causes gating defects [19].

The purpose of this study was to test in an in vitro model (intestinal organoids) the effect of all FDA-registered targeted drugs on restoring N1303K-*CFTR* channel function, and, in case of a positive effect, to conduct a comprehensive study of potential drugs, including an assessment of the *CFTR* function using ICM (ex vivo) and changes in the patient’s clinical parameters (in vivo).

## 2. Results

### 2.1. Combination of Three CFTR Modulators, VX-770, VX-661 and VX-445, Restores Chloride Channel Function in Intestinal Organoids’ Epithelium Derived from CF Patients with N1303K Variant

**Figure 1:**
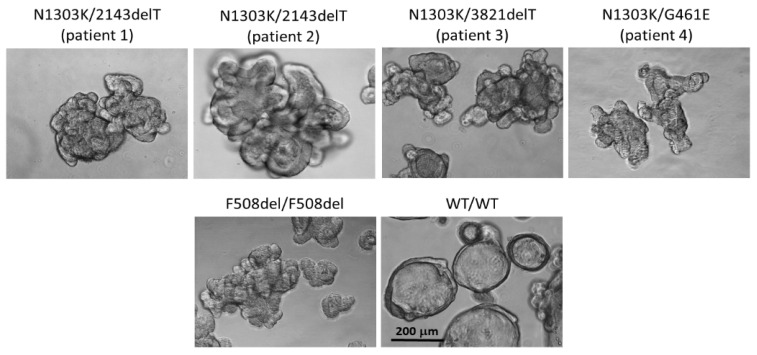
Morphology of intestinal organoids derived from CF patients with N1303Kvariant compared to c F508del/F508del and wt/wt *CFTR* cultures (controls). Five days in culture, 20× objective, scale bar: 200 μm.

To study the effects of *CFTR* modulators in vitro, we have established four intestinal organoid cultures compound heterozygous for *CFTR*: N1303K with class I variants 2143delT (two patients), 3821delT (one patient) and with the rare variant G461E (one patient). Variants 2143delT and 3821delT induce frameshifting and are classified as «severe»; G461E (p.Gly461Glu) is a rare pathogenic missense variant, which leads to the substitution of glycine for glutamine acid; the class of this variant is unknown. As a control of morphological features, we used intestinal organoids derived from a CF patient with the F508del/F508del genotype, as well as a healthy donor possessing the normal *CFTR* gene (wt/wt *CFTR*). The morphological features were similar in five CF patient-derived cultures bearing genotypes N1303K/2143delT (patient 1 and patient 2), N1303K/3821delT (patient 3), N1303K/G461E (patient 4) and F508del/F508del (control). All cultures are characterized by a reduced lumen and thickened walls (Figure 1). In contrast, wt/wt *CFTR* organoids due to a functional *CFTR* channel possess a pronounced lumen and thin walls.

Thus, morphological features of patient-derived organoid cultures with the N1303K variant confirm the absence of a functional *CFTR* protein.

**Figure 2:**
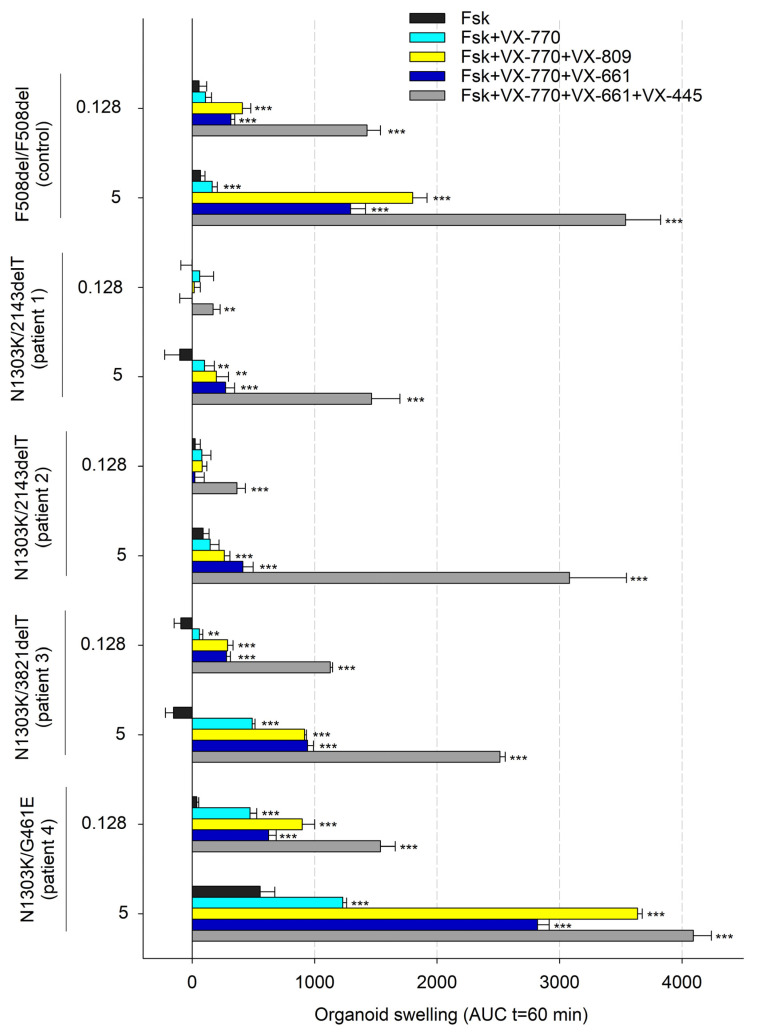
Assessment of *CFTR* modulators’ effects on forskolin (Fsk) treatment in CF organoids with N1303K variant. Control–F508del/F508del organoids; each modulator concentration is 3.5 µM; ** *p* < 0.01, *** *p* < 0.001 compared to Fsk alone by unpaired Student’s *t*-test.

**Figure 3:**
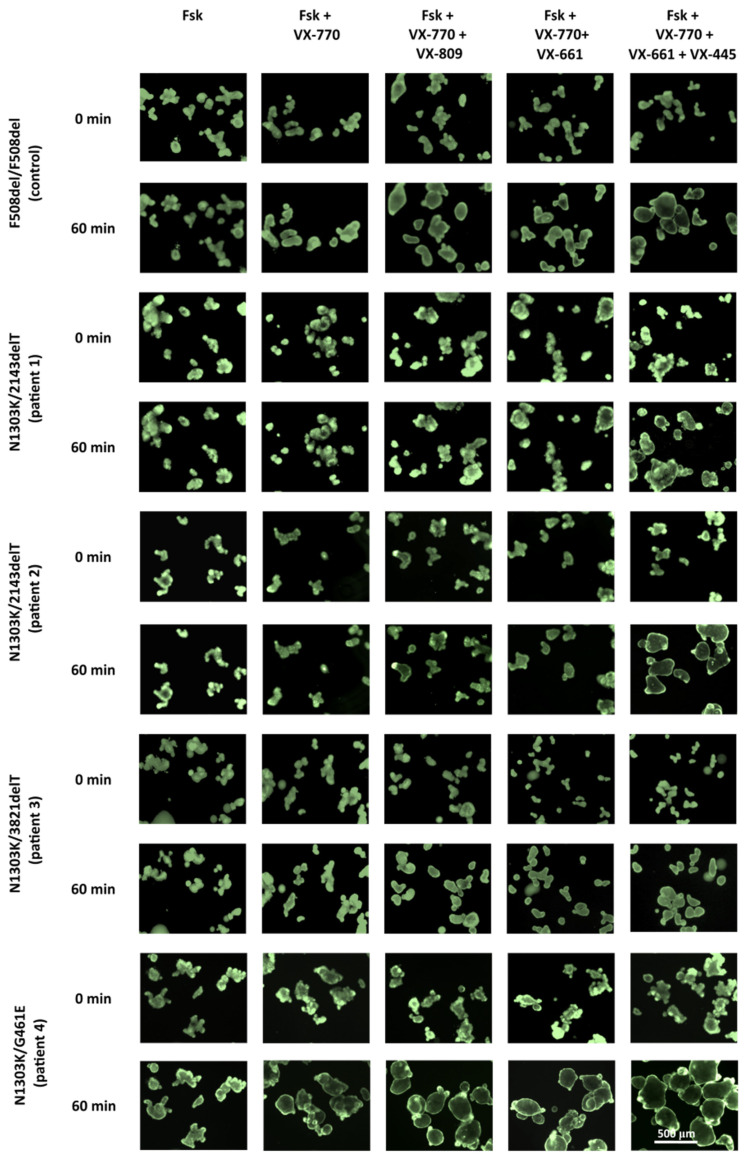
N1303K/2143delT, N1303K/3821delT, N1303K/G461E and F508del/F508del (control) intestinal organoid swelling upon 1 h treatment with 5 µM forskolin. Organoids were stained with Calcein AM, and each *CFTR*-modulator concentration is 3.5 µM; Scale bar: 500 μm.

Forskolin stimulation was used to evidence the *CFTR* function. The intensity of responses to forskolin treatment combined with *CFTR* modulators was used to show the influence of a potentiator and correctors on the restoration of *CFTR* function. Forskolin stimulation of N1303K/class I organoids (patients 1–3) induced no swelling as observed in the control culture (Figure 2 and Figure 3). Lack of response to forskolin application provides evidence for the complete loss of *CFTR* channel function. The presence of the rare variant G461E in the genotype is accompanied by high residual *CFTR* channel function since N1303K/G461E organoids respond with significant swelling upon forskolin stimulation (5 µM, 1 h). The response is 555 ± 120 arbitrary AUC units (area under the curve, a.u.) and the total organoids’ surface area increases by 25% compared to original area (Figure 2 and Figure 3).

Notably, the intensity of responses to forskolin treatment combined with *CFTR* modulators was different between the investigated cultures; the influence of the potentiator and correctors was considerably lower in organoids with the N1303K/2143deT genotype (from patients 1 and 2) compared to organoids with the N1303K/3821delT genotype (from patient 3). Upon treatment with 0.128 μM forskolin in combination with VX-770 + VX-809 or VX-770 + VX-661, no swelling was observed in N1303K/2143deT organoids, while N1303K/3821delT organoids showed weak responses, comparable to F508del/F508del control: ~300–400 a.u. Triple combination VX-770 + VX-445 + VX-661 induced significant (>1000 a.u.) responses in N1303K/3821delT organoids even upon 0.128 μM forskolin stimulation in contrast to both N1303K/2143delT cultures (Figure 2). For N1303K/G461E organoids with residual *CFTR* function, when exposed to 0.128 μM forskolin, a pronounced response to the potentiator and all three combined drugs was observed; organoids swelled due to an increase in the amount of functional *CFTR* protein.

To determine the potential therapeutic effect of targeted drugs in “severe” genotypes, forskolin is used in high (5 µM) concentration and organoids are treated for 1 h. For AUC values below 1000 arbitrary units, targeted drug treatment is not recommended, as it will not provide a significant therapeutic effect [22]. Under the indicated treatment conditions (5 µM Fsk, 1h), we have shown that the organoids with the N1303K variant are sensitive to the effects of VX-770 + VX-445 + VX-661. Patient 1’s (N1303K/2143delT) intestinal organoids showed a 50% volume expansion (AUC = 1465 ± 231 a.u.), while patients 2, 3 and 4’s organoids expanded more than a twofold volume expansion, and AUC values were 3080 ± 465, 2514 ± 43 and 4091 ± 148 a.u., respectively.

### 2.2. Elexacaftor + Tezacaftor + Ivacaftor Treatment of N1303K/2143delT Patient Produces a Positive Therapeutic Effect

Due to logistical and regulatory constraints, we could only evaluate the therapeutic applicability of our findings on patient 1, a 19-year-old male, to whom it was possible to prescribe the Elexacaftor + Tezacaftor + Ivacaftor (ETI) targeted therapy. The patient experienced pancreatic insufficiency from the first weeks of life, which was later accompanied by manifestations of bronchopulmonary infection. The diagnosis of CF was established at 10 months, sweat chlorides amounted to 84 and 89 mmol/L (sweat conductivity), and the diagnosis was subsequently confirmed by identification of two pathogenic variants of the *CFTR* gene: 2143delT and N1303K. From the age of 7 years, the patient experienced respiratory tract infection by *P. aeruginosa*. Basic therapy was carried out that included rhDNase, hypertonic saline, inhaled tobramycin and sodium colistimethate, pancreatic enzymes, azithromycin and vitamins according to general guidelines. Due to the absence of severe lung exacerbations and normal respiratory function, intravenous antibiotic therapy was unnecessary.

Before the start of ETI, November 2022 (Table 1), complaints included general fatigue; daily, sometimes increasing, unproductive cough; frequent episodes of discomfort and abdominal pain; unstable stool one–four times a day. During a physical examination, clean skin, normal physical status (height 180 cm, weight 70 kg, BMI 21.6 kg/m^2^, respiratory rate 18 per minute, normal lungs auscultation, SpO2 98%) were observed. The abdomen was soft and painless. There were no signs of oedema.

Upon CT scan of the chest, the chest shape was normal. In all pulmonary fields, mainly in the upper and middle sections of both lungs, dilation of III-IV order bronchi was observed along with multiple predominantly cylindrical bronchiectases and bronchiolectases.

Spirometric parameters were FVC 5.52 L (100.5% of predicted value), FEV_1_ 3.88 L (84.1% of predicted value) and FEV_1_/FVC 70.2%. The sweat test result was 103 mmol/L (sweat conductivity). The microbiological analysis of sputum revealed *S.aureus*, *P.aeruginosa*, *Rahnella* sp. and *Neisseria macacae.*

In December 2022, the treatment with ETI was initiated.

After 3 months from the start of therapy, March 2023 (Table 1), the patient’s parameters were as follows: weight—72 kg and BMI—22.2 kg/m^2^. Spirometric parameters were FVC 5.82 L (105.9% of predicted value), FEV_1_ 4.1 L (88.8% of predicted value) and FEV_1_/FVC 70.4%. The sweat test result was 117 mmol/L (sweat conductivity). In microbiological analysis of sputum, a decrease in the flora diversity was observed, namely *St. aureus* and *P. aeruginosa.*

Upon treatment, there was a noticeable decrease in daily cough frequency and improvement in sputum expectoration, lower fatigue and relief from abdominal discomfort with stool once a day.

### 2.3. Evaluation of the Effectiveness of Elexacaftor + Tezacaftor + Ivacaftor in a Patient with the N1303K/2143delT Genotype Using the Intestinal Current Measurement (ICM) Method

Before the start of ETI therapy and after 3 months of treatment, patient 1 with the N1303K/2143delT genotype was tested using ICM. ICM makes it possible to assess the functional activity of membrane channels ex vivo on rectal biopsy samples using selective channel agonists/activators or inhibitors. A positive response to forskolin stimulation indicates the presence of functional *CFTR*.

**Figure 4:**
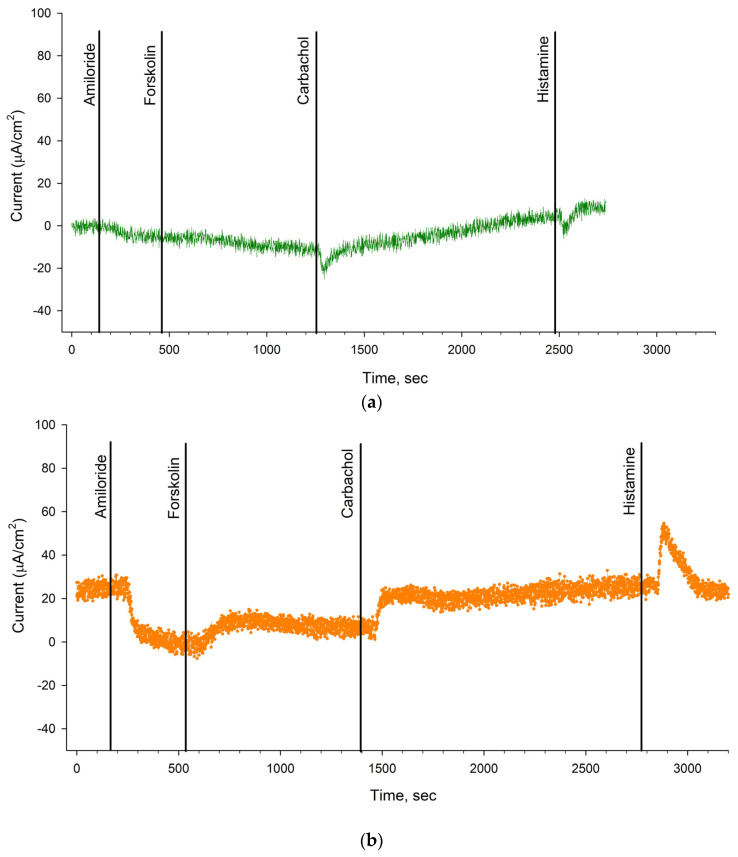
Results of functional activity study of intestinal epithelial ion channels in N1303K/2143deT patient by ICM: (**a**) before the treatment with targeted therapy, there is no response to forskolin and a negative change of ΔI_SC_ upon carbachol and histamine application; (**b**) during the targeted therapy and 3 months later, a positive changed ΔI_SC_ was observed after forskolin, carbachol and histamine application. The plots in Figure 4a,b are representative, and each plot is the result of a short-circuit current measurement for one of the three studied biopsies.

It was shown that before treatment with ETI (Table 2, Figure 4a), the change in short-circuit current density (ΔI_SC_) in response to amiloride administration (ENaC inhibitor) was −7.0 ± 3.18 µA/cm^2^. The change in ΔI_SC_ in response to forskolin stimulation (*CFTR* activation) was 4.33 ± 2.51 µA/cm^2^, which was significantly lower than in the negative control group (wt/wt *CFTR*; 26.72 ± 2.66) and almost no different from to the positive control (F508del/F508del; 3.33 ± 0.63). In response to carbachol administration (activation of CaCCs), ΔI_SC_ showed negative changes reaching 10.0 ± 2.47 µA/cm^2^ (Table 2). In response to histamine (activation of CaCCs), ΔI_SC_ also changed in the negative direction (Figure 4a) and reflected the influx of potassium ions into the cells, which is typical for patients with CF. The current density was 8.17 ± 1.24 µA/cm^2^ (Table 2) and was significantly lower than the wt/wt *CFTR* values of the negative control (109.76 ± 8.18). These results provide evidence for the absence of a functional *CFTR* channel in the patient bearing N1303K and 2143delT pathogenic variants.

In the course of ETI treatment (Table 2, Figure 4b), ΔI_SC_ in response to amiloride (ENaC inhibitor) application was −11.67 ± 6.09 µA/cm^2^. ΔI_SC_ change upon forskolin application reached 8.33 ± 3.47 µA/cm^2^, which was higher compared to the values before therapy (Table 2). In response to carbachol administration, ΔI_SC_ achieved 15.17 ± 0.2 µA/cm^2^, which was higher compared to the values before the therapy was initiated. In response to histamine administration, ΔI_SC_ changed in a positive direction and achieved 15.67 ± 3.54 µA/cm^2^. The results show restoration of *CFTR* channel function in the patient during therapy.

## 3. Discussion

For the first time, a comprehensive study was conducted on an example of one patient with the N1303K/class I genotype to examine the ETI effect on the restoration of *CFTR* function using ex vivo (ICM), in vitro (patient’s intestinal organoids) and assessment of clinical parameters before and three months after treatment with ETI. All obtained results are consistent with each other and have proven the effectiveness of ETI for the N1303K variant.

A study of the therapeutic effect of ETI involving eight patients with the N1303K variant was performed by I. Sadras et al. in 2023 [20]. The authors examined the effects of targeted therapy on lung function, BMI and sweat chloride levels. Studies were also performed on the intestinal organoids of six patients. The results of the study by I. Sadras et al. on organoids indicate a moderate response (AUC is 200–800 a.u.) to the effects of ETI, in contrast to our data (AUC for three cultures bearing N1303K/class I genotypes is ~1500–3100 a.u.).

We have shown that the N1303K/class I organoid size increases by 1.5–2 times at the end of the FIS assay. Of all cultures, the N1303K/G461E organoids were the most sensitive to *CFTR* modulator administration, likely primarily due to the rare G461E missense variant. Thus, using an intestinal organoids model, we have demonstrated that the N1303K-*CFTR* protein function is restored by the combined effect of ETI.

The obtained results are in line with previously published results where a combination of ETI restores *CFTR* function in intestinal organoids bearing the N1303K variant [13,23]. In a 2022 study by Ensinck et al., using an FIS assay on intestinal organoids with the N1303K variant, a positive effect of ETI was demonstrated, which was expressed in a significant swelling of organoids upon treatment with forskolin and Ivacaftor after a preceding 24 h incubation with an Elexacaftor + Tezacaftor combination [24]. Thus, the significant pharmacological response observed in N1303K/2143delT and N1303K/3821delT intestinal organoid cultures laid the basis for prescribing the combined ETI therapy to the patient with the N1303K/class I. Notably, our FIS assay data also offered valuable insights discouraging the prescription of VX-770 + VX-809 (Ivacaftor + Lumacaftor) or VX-770 + VX-661 (Ivacaftor + Tezacaftor) therapy for N1303K/class I patients.

In the study of I. Sadras et al. [20], despite the moderate organoid response, patients experienced significant clinical improvement. The increase in FEV_1_ after 2 months (8 weeks) of ETI ranged from 9 to 30% (mean increase in FEV_1_ across eight patients amounted to 18%). In another study of Elson et al. [23], the 1.5-month administration of ETI to an N1303K patient resulted in a 9% increase in FEV_1_. Furthermore, Y. Huang et al. [25] have demonstrated that the ETI treatment of an 11-year-old N1303K/E193X patient for 10 months had improved his FEV_1_ from 87% to 108% (21% total). In our patient, FEV_1_ was improved by 4.7% (from 84.1% to 88.8% predicted) after a 3-month treatment.

After 3 months of ETI therapy, the BMI increased by 0.6 kg/m^2^ (body weight gain was 2.0 kg). According to Elson et al. [23], 12-month ETI administration to the N1303K patient increased his BMI from the 29th to the 38th percentile (body weight increased by 2.9 kg), while according to Y. Huang [25] 10-month ETI treatment increased the BMI from the 34.8th to the 45th percentile (body weight increased by 4.9 kg). Finally, Sadras et al. [20] have shown that ETI administration in eight patients produced a mean BMI increase of 0.79 kg/m^2^.

Sweat test results obtained in our study did not reveal a decrease in the concentration of sweat chlorides (before therapy—103 mmol/L, after 3 months of therapy—117 mmol/L, while normal levels are below 30 mmol/L or below 50 mmol/L if conductivity test is used), which is in accordance with previously published data [20,23,25]. Thus, Huang et al. [25] have shown that sweat chloride levels were only marginally lowered in a female patient by ETI administration (from 108 mmol/L before treatment to 95 mmol/L after the treatment). According to Elson et al. [23], after 2 months of ETI treatment the patient’s sweat chloride levels remained elevated: 113 mmol/L and 111 mmol/L. Ultimately, in Sadras et al.’s study [20] of eight patients with the N1303K variant, triple drug therapy caused only a slight decrease in sweat chloride concentration, with their values exceeding the norm by three or more times.

We have noted an improvement in the microbial landscape without the suppression of lung pathogenic microflora in contrast to Huang’s data [25], who observed both pathogenic microflora suppression and improvement in the microbial landscape after 10 months of ETI therapy.

Thus, the results of our investigation in a N1303K/2143delT patient are generally consistent with previous data [20,23,25]: ETI treatment does not affect the sweat chloride levels while producing a significant improvement in lung function and nutritional status. Previously, it was assumed [20] that such unexpected results may be explained by the differential efficiency of ETI in airway, gastrointestinal and sweat gland epithelial cells, or by varying degrees of restoration of the N1303K-*CFTR* processing depending on the target tissue.

Assessment of the functional activity of the N1303K/2143delT patient’s intestinal epithelium ion channels using ICM showed positive dynamics in restoring *CFTR* chloride channel function in the course of the targeted therapy treatment. The results using ICM proving ex vivo the positive therapeutic effect of ETI were obtained for the first time. The obtained data are consistent with the FIS assay results as well as respiratory function parameters, abdominal discomfort relief and general improvement in the patient’s condition, but they do not correlate with the sweat test results.

The comprehensive approach used in this work allows us to obtain convincing evidence for the therapeutic effectiveness of *CFTR* modulators. However, the obtained results do not allow us to explain the lack of *CFTR* channel response in the sweat gland epithelium in patients with the N1303K variant receiving ETI. Our data support the idea that sweat chloride concentration may not be an ideal biomarker to assess response to ETI at the functional level in patients with the N1303K variant. More extensive research including more patients and additional molecular investigations is needed to correlate in vitro data with clinical outcomes in patients with N1303K.

## 4. Materials and Methods

This study involved four CF patients with the N1303-*CFTR* gene variant. Intestinal organoid cultures were obtained from all four patients, and for one of the patients with the N1303K/2143delT genotype, a comprehensive study of ETI therapeutic effect was conducted, including ICM and assessment of the clinical status. The study involved 18 healthy volunteers who formed a wt/wt control group for ICM; a control culture of intestinal organoids was obtained from one of the volunteers. The study involved five CF patients bearing the F508del/F508del genotype, who formed the CF control group for ICM; a control culture of intestinal organoids was obtained from one patient with the F508del/F508del genotype.

All participants took part in this study after signing the informed voluntary consent form. This form, based on the principles of the Declaration of Helsinki, was approved by the Ethics Committee of Research Centre for Medical Genetics, Ministry of Science and Education, Russian Federation on 15 October 2018 (Ethics Committee chairman—Prof. L.F. Kurilo). Off-label treatment with ETI was carried out under medical supervision at the Pulmonology Scientific Research Institute under the Federal Medical and Biological Agency of the Russian Federation.

### 4.1. Forskolin-Induced Swelling (FIS) Assay in Intestinal Organoids

For this study, long-term intestinal organoid cultures with wt/wt *CFTR*, F508del/F508del, N1303K/2143delT, N1303K/3821delT and N1303K/G461E genotypes were established. Organoid derivation and an FIS assay were based on protocols of J. Beekman [26].

Mouse L-fibroblast line, transfected with Wnt-3A-expression vector, and HEK293 line, transfected with Noggin-expression vector for preparation of conditioned media with corresponding growth factors, were kindly provided by J. Beekman [26]. HEK293T–R-spondin-1 mFc line, transfected with a corresponding expression vector was used as a source of R-spondin-1 (SCC111, Merck Millipore S.A.S., Molsheim, France). Conditioned media enriched with growth factors were prepared using DMEM + GlutaMax medium (Thermo Fisher Scientific, Waltham, MA, USA), Advanced DMEM/F12 medium (Thermo Fisher Scientific, Waltham, MA, USA), fetal bovine serum (Thermo Fisher Scientific, Waltham, MA, USA), penicillin/streptomycin (PanEco, Moscow, Russia), zeocin (Thermo Fisher Scientific, Waltham, MA, USA), G418 (PAA Laboratories, Pasching, Austria), 1 M HEPES-Na (PanEco, Moscow, Russia), L-glutamine (PanEco, Moscow, Russia), PBS (PanEco, Moscow, Russia), 0.25% trypsin (PanEco, Moscow, Russia), TrypLE Express (Waltham, MA, USA), T175 cell culture flasks with air filters (Corning, Corning, NY, USA), 150 mm Petri dishes (SPL Life Sciences, Yeoju-si, Korea), serological pipettes (SPL Life Sciences, Yeoju-si, Korea) and 15 and 50 mL sterile centrifuge tubes (SPL Life Sciences, Yeoju-si, Korea). Detailed description of the conditioned media preparation is provided in [26].

Organoid culture medium contained Wnt-3A-, Noggin- and R-spondin-1 conditioned media (50%, 10% and 20%, respectively), Advanced DMEM/F12 (18%; Thermo Fisher Scientific, Waltham, MA, USA), mEGF (50 ng/mL; Prospec, Ness-Ziona, Israel), B27 Supplement (2%; Thermo Fisher Scientific: Gibco, Waltham, MA, USA), *N*-acetyl cysteine (1.25 mM; Sigma-Aldrich, St. Louis, MO, USA), nicotinamide (10 mM; Sigma-Aldrich, St. Louis, MO, USA), A83-01 (5 µM; Sigma-Aldrich, St. Louis, MO, USA), SB 202190 (10 µM; Sigma-Aldrich, St. Louis, MO, USA) and Primocin (100 μg/mL; InvivoGen, San Diego, CA, USA).

Crypts were obtained from rectal biopsies, and organoids were passaged as described [14,26].

Three biopsy samples were collected from each CF patient and one healthy donor. Biopsies were consecutively washed with Advanced DMEM/F12 and PBS, then treated with 10 mM EDTA solution (Thermo Fisher Scientific: Invitrogen, Waltham, MA, USA). The obtained material was resuspended after EDTA treatment to liberate the individual crypts from the tissue. The precipitated crypts were then mixed with Matrigel^®^ (Corning, Bedford, MA, USA) and seeded on 24-well culture plates (SPL Life Sciences, Yeoju-si, Korea). Organoids were passaged once per week. For that purpose, the culture medium from all wells was removed, Matrigel droplets were disrupted, and organoids were mechanically dissociated into smaller fragments. The obtained suspension was centrifuged for 5 min at 130 g and 4 °C. Precipitated organoids were mixed with Matrigel^®^ and seeded on 24-well culture plates. Medium exchange was performed every 2–3 days.

Evaluation of target drugs’ effects on intestinal organoids was performed by an FIS assay. One day before the assay the organoids were seeded into Matrigel^®^ droplets into 96-well plates (SPL Life Sciences, Yeoju-si, Korea), and corresponding correctors VX-809 (Lumacaftor), VX-661 (Tezacaftor), VX-445 (Elexacaftor) (all at 3.5 μM concentration; Selleckchem, Houston, TX, USA) were added at this timepoint. Forskolin (Sigma-Aldrich, St. Louis, MO, USA) treatment was preceded by 30 min Calcein staining (0.85 μM; BioLegend, San Diego, CA, USA). VX-770 potentiator (3.5 μM; Selleckchem, Houston, TX, USA) was added simultaneously with forskolin (0.128 or 5 μM). The intestinal organoids were incubated with forskolin and the *CFTR* modulators for 1 h, and the selected fields of view were imaged with 10 min intervals using Axio Observer 7 Fluorescence microscope (Zeiss, Oberkochen, Germany). Quantitative analysis of organoid swelling was performed using Image J (v1.52n state version, NCBI, Bethesda, MD, USA) and Sigma Plot 12.5 (Systat Software, Inc., Chicago, IL, USA) software. Statistical data analysis was performed using Microsoft Excel (Microsoft Corporation, Redmond, WA, USA). The organoid swelling results are expressed as the absolute area under the curve (AUC) calculated from the normalized increase of the surface area (baseline = 100%, t = 60 min). In this study, organoid cultures from the F508del/F508del (c.1521_1523delCTT/c.1521_1523delCTT) patient and healthy donor (wt/wt *CFTR*) were used for validation. All cell cultures were maintained at 37 °C in a humidified air atmosphere containing 5% CO_2_.

### 4.2. Intestinal Current Measurement (ICM)

Before the start of ETI therapy and after three months of treatment, patient 1 with the N1303K/2143delT genotype was tested using ICM. The study using the ICM was conducted according to the European Standard operating procedures V2.7_26.10.11 (SOP) [27]. At the first stage, each recirculation chamber was calibrated separately on the VCC MC 8B421 Physiological Instrument (San Diego, CA, USA). At the second stage, after calibration of the device, rectal biopsy material was placed in the chamber. Biopsy samples were collected using Olympus Disposable EndoTherapy En-doJaw Biopsy forceps equipment (model #FB-23OU, Tokyo, Japan) according to manufacturer guidelines. Three biopsy samples were collected from each patient. The size of the biopsy was 3–5 mm. The biopsy material was placed in a special slider, which was then inserted into the recording camera. The chambers were filled with Meyler buffer solution. The buffer was prepared immediately before the study and consisted of 105 mM NaCl, 4.7 mM KCl, 1.3 mM CaCl_2_·6H_2_O, 20.2 mM NaHCO_3_, 0.4 mM NaH_2_PO_4_·H_2_O, 0.3 mM Na_2_HPO_4_, 1.0 mM MgCl_2_·6H_2_O, 10 mM HEPES and 10 mM D-glucose, as well as 0.01 mM indomethacin. The study began with the recording of the basal short-circuit current (µA/cm^2^) (pre-amiloride stage). At the third stage, compounds were added in the following sequence: amiloride (final 100 μM), forskolin (final 10 μM), carbachol (final 100 μM) and, at the end, histamine (500 μM). All reagents used were from Sigma-Aldrich (Merck, Darmstadt, Germany). The negative control group consisted of healthy volunteers. The positive control group consisted of patients homozygous for the F508del variant (F508del/F508del). The control group consisted of 18 people; group F508del/F508del of 5 people.

### 4.3. Description of the Clinical Picture

The following characteristics were assessed in patients with the N1303K/2143delT genotype before and 3 months after the start of ETI: age at diagnosis, age at the time of the study, nutritional status (BMI), clinical characteristics and complaints, sweat test results (conductivity sweat test), sputum microbiology (standard microbiological culture and phenotypic identification of Gram-positive and Gram-negative bacteria in sputa), lung function (percentage predicted FEV_1_, FVC and the FEV_1_/FVC ratio), pancreatic function as use of PERT and other symptomatic therapy.

### 4.4. Statistical Analyses

The FIS results for each intestinal organoid culture are presented as mean AUC ± SD, with the number of biological (3) and technical (4–6) replicates. Statistical analysis was performed by unpaired two-tailed Student’s *t*-test. The ICM study was carried out on 3 biopsy samples for each patient; in Table 2, the results are presented as M ± m per study.

## 5. Conclusions

A comprehensive assessment of the N1303K variant using three approaches (ICM, a patient’s intestinal organoids and evaluation of clinical parameters) showed that the VX-445 + VX-661 + VX-770 (ETI) combined effect induces pharmacological response and restores N1303K-*CFTR* function. ETI produced a significant positive effect on forskolin-induced swelling in N1303K/class I organoids, indicating the functional improvement of the *CFTR* protein. The results obtained in vitro provided the basis for prescribing targeted therapy to the N1303K/2143delT patient. ETI treatment for 3 months contributed to stabilization of clinical status, BMI increase, improvement in lung function and decreased diversity of lung microbial pathogens, while it produced no effect on sweat chloride concentration. Intestinal current measurement confirmed the restoration of *CFTR* channel function. FIS assay data also offered valuable insights discouraging the prescription of VX-770 + VX-809 (Ivacaftor + Lumacaftor) or VX-770 + VX-661 (Ivacaftor + Tezacaftor) therapy for N1303K/class I patients.

## Figures and Tables

**Table 1:** Main clinical data of a patient with the N1303K/2143delT genotype before and 3 months after starting treatment with ETI.

FEV_1_% Predicted			BMI (kg/m^2^)			Sweat Chloride, mmol/L (Conductivity)		
Before	After	Change	Before	After	Change	Before	After	Change
84.1	88.8	4.7	21.6	22.2	0.6	103	117	14

**Table 2:** Short-circuit current density (µA/cm^2^) in the Ussing chamber before targeted therapy and 3 months after the treatment in comparison with the positive and negative control groups.

	Amiloride, 100 μM	Forskolin, 10 μM	Carbachol, 100 μM	Histamine, 500 μM
N1303K/2143delT before ETI therapy	−7.0 ± 3.18	4.33 ± 2.51	10.0 ± 2.47	8.17 ± 1.24
N1303K/2143delT 3 months after ETI therapy	−11.67 ± 6.09	8.33 ± 3.47	15.17 ± 0.2	15.67 ± 3.54
positive control (F508del/F508del)	−15.7 ± 3.51	3.33 ± 0.63	-	17.83 ± 3.57
negative control (wt/wt *CFTR*)	−7.67 ± 1.76	26.72 ± 2.66	117.44 ± 4.32	109.76 ± 8.18

## Data Availability

The datasets used and/or analysed during the current study are available from the corresponding author upon reasonable request.

## References

[1] Castellani C., Duff A.J.A., Bell S.C., Heijerman H.G.M., Munck A., Ratjen F., Sermet-Gaudelus I., Southern K.W., Barben J., Flume P.A. (2018). ECFS Best Practice Guidelines: The 2018 Revision. J. Cyst. Fibros..

[2] Lopes-Pacheco M. (2020). CFTR Modulators: The Changing Face of Cystic Fibrosis in the Era of Precision Medicine. Front. Pharmacol..

[3] Bacalhau M., Camargo M., Magalhães-Ghiotto G.A., Drumond S., Castelletti C.H., Lopes-Pacheco M. (2023). Elexacaftor-Tezacaftor-Ivacaftor: A Life-Changing Triple Combination of CFTR Modulator Drugs for Cystic Fibrosis. Pharmaceuticals.

[4] Cystic Fibrosis Foundations. https://www.cff.org/managing-cf/cftr-modulator-therapies.

[5] Mergiotti M., Murabito A., Prono G., Ghigo A. (2022). CFTR Modulator Therapy for Rare CFTR Mutants. J. Respir..

[6] Costa E., Girotti S., Pauro F., Leufkens H.G.M., Cipolli M. (2022). The Impact of FDA and EMA Regulatory Decision-Making Process on the Access to CFTR Modulators for the Treatment of Cystic Fibrosis. Orphanet J. Rare Dis..

[7] Ghorbaninejad M., Asadzadeh-Aghdaei H., Baharvand H., Meyfour A. (2023). Intestinal Organoids: A Versatile Platform for Modeling Gastrointestinal Diseases and Monitoring Epigenetic Alterations. Life Sci..

[8] Dekkers J.F., Wiegerinck C.L., de Jonge H.R., Bronsveld I., Janssens H.M., de Winter-de Groot K.M., Brandsma A.M., de Jong N.W.M., Bijvelds M.J.C., Scholte B.J. (2013). A Functional CFTR Assay Using Primary Cystic Fibrosis Intestinal Organoids. Nat. Med..

[9] Dekkers J.F., Berkers G., Kruisselbrink E., Vonk A., De Jonge H.R., Janssens H.M., Bronsveld I., Van De Graaf E.A., Nieuwenhuis E.E.S., Houwen R.H.J. (2016). Characterizing Responses to CFTR-Modulating Drugs Using Rectal Organoids Derived from Subjects with Cystic Fibrosis. Sci. Transl. Med..

[10] Saini A. (2016). Cystic Fibrosis Patients Benefit from Mini Guts. Cell Stem Cell.

[11] Kondratyeva E., Efremova A., Melyanovskaya Y., Voronkova A., Polyakov A., Bulatenko N., Adyan T., Sherman V., Kovalskaia V., Petrova N. (2022). Evaluation of the Complex p.[Leu467Phe;Phe508del] CFTR Allele in the Intestinal Organoids Model: Implications for Therapy. Int. J. Mol. Sci..

[12] Furstova E., Dousova T., Beranek J., Libik M., Fila L., Modrak M., Cinek O., Macek M., Drevinek P. (2022). Response to Elexacaf Tor/Tezacaf Tor/Ivacaf Tor in Intestinal Organoids Derived from People with Cystic Fibrosis. J. Cyst. Fibros..

[13] Lefferts J.W., Bierlaagh M.C., Kroes S., Nieuwenhuijze N.D.A., Van Kooten H.N.S., Niemöller P.J., Verburg T.F., Janssens H.M., Muilwijk D., Van Beuningen S.F.B. (2023). CFTR Function Restoration upon Elexacaftor/Tezacaftor/Ivacaftor Treatment in Patient-Derived Intestinal Organoids with Rare CFTR Genotypes. Int. J. Mol. Sci..

[14] Kondratyeva E., Efremova A., Melyanovskaya Y., Petrova N., Satsuk N., Bulatenko N., Bukharova T., Zodbinova A., Sherman V., Kashirskaya N. (2020). Clinical and Genetic Characterization of Patients with Cystic Fibrosis and Functional Assessment of the Chloride Channel with the Pathogenic Variant c.831G>A (p.Trp277*), Described for the First Time. Gene.

[15] Kondratyeva E., Bukharova T., Efremova A., Melyanovskaya Y., Bulatenko N., Davydenko K., Filatova A., Skoblov M., Krasovsky S., Petrova N. (2021). Health Characteristics of Patients with Cystic Fibrosis Whose Genotype Includes a Variant of the Nucleotide Sequence c.3140-16t>a and Functional Analysis of This Variant. Genes.

[16] Kondratyeva E.I., Krasovsky S.A., Starinova M.A., Voronkova A.Y., Amelina E.L., Kashirskaya N.Y., Avdeev S.N., Kutsev S.I. (2022). Register of Patients with Cystic Fibrosis in the Russian Federation, 2020 Year.

[17] Van Rens J., Prasad V., Fox A., Krasnyk M., Mayor S.L., Naehrlich L., Gkolia P., Nieto A.E., Bakkeheim E., Daneau G. (2023). ECFSPR Annual Report 2021. https://www.ecfs.eu/sites/default/files/Annual%20Report_2021_09Jun2023_ECFSPR_final.pdf.

[18] (2022). Cystic Fibrosis Foundation Patient Registry 2021 Annual Data Report; Bethesda, Maryland. https://www.cff.org/sites/default/files/2021-11/Patient-Registry-Annual-Data-Report.pdf.

[19] DeStefano S., Gees M., Hwang T.-C. (2018). Physiological and Pharmacological Characterization of the N1303K Mutant CFTR. J. Cyst. Fibros..

[20] Sadras I., Kerem E., Livnat G., Sarouk I., Breuer O., Reiter J., Gileles-Hillel A., Inbar O., Cohen M., Gamliel A. (2023). Clinical and Functional Efficacy of Elexacaftor/Tezacaftor/Ivacaftor in People with Cystic Fibrosis Carrying the N1303K Mutation. J. Cyst. Fibros..

[21] Zhang Z., Chen J. (2016). Atomic Structure of the Cystic Fibrosis Transmembrane Conductance Regulator. Cell.

[22] Ramalho A.S., Förstová E., Vonk A.M., Ferrante M., Verfailli C., Dupont L., Boon M., Proesmans M., Beekman J.M., Sarouk I. (2021). Correction of CFTR Function in Intestinal Organoids to Guide Treatment of Cystic Fibrosis. Eur. Respir. J..

[23] Elson E.C., Capel P., Haynes J., Duehlmeyer S., Fischer M., Escobar H. (2022). CFTR Modulator Therapy in an Individual with Cystic Fibrosis Caused by a N1303K CFTR Variant and Infected with Mycobacterium Abscessus. J. Pediatr. Pharmacol. Ther..

[24] Ensinck M.M., De Keersmaecker L., Ramalho A.S., Cuyx S., Van Biervliet S., Dupont L., Christ F., Debyser Z., Vermeulen F., Carlon M.S. (2022). Novel CFTR Modulator Combinations Maximise Rescue of G85E and N1303K in Rectal Organoids. ERJ Open Res..

[25] Huang Y., Paul G., Lee J., Yarlagadda S., McCoy K., Naren A.P. (2021). Elexacaftor/Tezacaftor/Ivacaftor Improved Clinical Outcomes in a Patient with N1303K-CFTR Based on In Vitro Experimental Evidence. Am. J. Respir. Crit. Care Med..

[26] Vonk A.M., van Mourik P., Ramalho A.S., Silva I.A.L., Statia M., Kruisselbrink E., Suen S.W.F., Dekkers J.F., Vleggaar F.P., Houwen R.H.J. (2020). Protocol for Application, Standardization and Validation of the Forskolin-Induced Swelling Assay in Cystic Fibrosis Human Colon Organoids. STAR Protoc..

[27] Derichs N., Sanz J., Von Kanel T., Stolpe C., Zapf A., Tümmler B., Gallati S., Ballmann M. (2010). Intestinal Current Measurement for Diagnostic Classification of Patients with Questionable Cystic Fibrosis: Validation and Reference Data. Thorax.

